# Optimization of Saffron Essential Oil Nanoparticles Using Chitosan-Arabic Gum Complex Nanocarrier with Ionic Gelation Method

**DOI:** 10.1155/2022/4035033

**Published:** 2022-03-07

**Authors:** Fitri Astutiningsih, Sri Anggrahini, Aprilia Fitriani, Supriyadi Supriyadi

**Affiliations:** ^1^Department of Food Technology and Agricultural Products, Faculty of Agricultural Technology, Universitas Gadjah Mada, Flora Street No. 1, Sleman, Special District of Yogyakarta 55281, Indonesia; ^2^Food Technology, Slamet Riyadi University, Sumpah Pemuda Street No. 18, Kadipiro, Surakarta, Central Java 57136, Indonesia; ^3^Food Technology, Faculty of Industrial Technology, Universitas Ahmad Dahlan, Jenderal Ahmad Yani Street, Banguntapan, Bantul, Special District of Yogyakarta, Indonesia

## Abstract

This study is aimed at optimizing the Saffron essential oil (SEO) nanoparticles using the ionic gelation method. Response surface methodology (RSM) with Box-Behnken design (BBD) was applied to investigate the optimum conditions and the effects of three independent variables: LWCS concentration (0.1-0.3%), Arabic gum concentration (9.6-9.8%), and ratio (core: wall material) (1 : 5, 1 : 7.5, 1 : 10) on the responses of *z*-average, polydispersity index (PDI), and zeta potential. The results showed that the quadratic model developed from the RSM was statistically significant (*p* value < 0.05). The quadratic model can be used to describe well the relationship between the variables on the response observed. The lack of fit was nonsignificant (*p* value > 0.05) relative to pure error for all response variables, indicating that the model fitted well. The model equation obtained for the process through RSM was adequate. The LWCS concentration and Arabic gum concentration had a significant effect on *z*-average and PDI. The ratio (oil: Arabic gum/LWCS) has a significant effect on zeta potential. The optimum condition was the LWCS concentration of 0.1% and Arabic gum concentration of 9.6%, and the ratio (oil: Arabic gum/LWCS) 1 : 5 produced the optimum SEO nanoparticles with a *z*-average value of 16.24, PDI of 0.495, and zeta potential of 15.76. The verification values were close to the predictive value given by the Design Expert® 12 program with *p* value > 0.05 at the 95% confidence level. Therefore, the application of the RSM with Box-Behnken was suitable for optimizing the saffron oil nanoparticles with desirable responses.

## 1. Introduction

Saffron (*Crocus sativus* L.) is the most expensive spice in the world [1]. Saffron is a rich source of rare C_20_ apocarotenoids with a variety of functional properties. These apocarotenoids are crocins, picrocrocin, and safranal [2]. Food industries extensively use saffron essential oil (SEO) as coloring and flavoring for foods [3]. SEO can be incorporated in food products, drinks, and beverages for its antimicrobial, antioxidant, and flavoring properties to improve the quality [4, 5]. However, SEO still faces low stability, susceptibility to oxidation (heat, oxygen, and light), and loss of flavor during processing and storage [6]. The stability of bioactive compounds in SEO can be improved and protected by converting them into saffron essential oil nanoparticles.

Among the various methods developed to prepare saffron essential oil nanoparticles, the ionic gelation technique has attracted considerable attention because this process is nontoxic, organic solvent-free, convenient, and controllable [7]. It is based on the interactions between the positively charged amino group of chitosan and the negatively charged groups of a polyanion [8]. These interactions form a polyelectrolyte complex bond on the particle surface, increasing the mechanical strength of the particles [9, 10]. Hosseini et al. [11] proposed nanoencapsulation of oregano EO by ionic gelation technique using chitosan nanoparticles. The data shows a *z*-average ranging from 10 to 60 nm and encapsulation efficiency of 77.8%. Then, Jamil et al. [12] prepared cardamom EO nanoparticles with chitosan using the ionic gelation technique. They formed the nanoparticles in a size range of 50–100 nm.

The choice of wall material is critical because it can affect the various attributes of the nanoparticles, including particle size, encapsulation efficiency, stability of bioactive compounds during storage, and food release [13, 14]. Several wall materials have been widely used for nanoparticles such as Arabic gum for saffron bioactive compound [15], low molecular weight chitosan (LWCS) for insulin [16], maltodextrin for saffron petal's anthocyanins [17], pectin, and whey for saffron extract encapsulation [18].

Low molecular weight chitosan (LWCS) is a class of third-generation biopolymers obtained from modifying chitosan through a depolymerization process to produce polymers with lower molecular weight and water-soluble [19]. LWCS can be used as a coating because it is food grade, nontoxic, and biodegradable. Due to the ionization of the amine groups of this biomolecule in acidic pH values, chitosan becomes water-soluble and positively charged. This feature provides a basis for the reaction of chitosan with other negatively charged macromolecules such as Arabic gum, carboxymethyl cellulose, alginate, and heparin, resulting in the formation of complexed nanoparticles. Various reports have been presented about the reaction of LWCS with different biopolymers/ions to prepare nanoparticles such as LWCS and Arabic gum for saffron extract [20] LWCS and alginate for mangosteen microparticles [21]. Fan et al. [22] prepared monodisperse low molecular weight chitosan nanoparticles with ionic gelation. The optimized nanoparticles exhibited a particle size range of 138 nm with a polydispersity index (PDI) of 0.026 and a zeta potential of +35 mV. Thus, in this study, we choose to use LWCS with a high degree of deacetylation.

Arabic gum is a nontoxic and polyanion hydrophilic biopolymer. The compact structure of Arabic gum gets easily opened in neutral pH values due to the dissociation of its carboxyl groups, resulting in a high level of negatively charged groups [23]. This was providing a suitable reaction with chitosan during ionic gelation [24]. Therefore, LWCS when mixed with Arabic gum is very good at forming polyelectrolyte complexation. It is supposed that the electrostatic complex between LWCS and Arabic gum could form strong viscoelastic films around oil droplets and provide them with a physical-chemical barrier against oxidation [25]. Avadi et al. [26] study develops insulin nanoparticles using chitosan and Arabic gum with an ionic gelation method. The developed nanoparticles showed the *z*-average between 172 and 205 nm, PDI 0.26, and zeta potential 41.2 mV.

An optimization process of formulation parameters is required to obtain the optimum condition of saffron essential oil nanoparticles. Since many parameters need to be studied when preparing the optimum nanoparticles, conventional experimental designs for optimizing all parameters are costly and time-consuming. Alternatively, numerical methods such as response surface methodology (RSM) can minimize the difficulties of conventional experimentation [27]. RSM is a statistical and mathematical approach that uses a second-degree polynomial model to study the relationship between one or more response variables and several independent variables using univariate or multivariate models [28]. The optimum conditions are obtained by using complex experimental designs including three-level full factorial design, central composite designs (CCD), Box-Behnken design (BBD), or Doehlert matrix (DM). BBD is one of the multivariate optimization techniques based on three-level incomplete factorial designs which were used for the second-order response surface model. According to Ferreira et al. and Klaus et al., BBD is superior to other response surface designs (e.g., three-level full factorial design, CCD, and DM) because it has no axial point, which contributes to the accomplishment of fewer costs of experimentation and reduces the experimental runs in analytical optimization. RSM has been widely used for optimizing the formulation of red palm oil nanoemulsions [29], D-limonene nanoencapsulation [30], fucoxanthin-rich oil nanoemulsion [31], and coconut shell liquid smoke nanoencapsulation [32].

This study is aimed at determining the optimal conditions of SEO nanoparticles based on the parameters of low molecular weight chitosan (LWCS) concentration, Arabic gum concentration, and the ratio between (core: wall material). Optimization is done by using the response surface methodology based on Box-Behnken design.

## 2. Materials

Saffron essential oil was purchased from RV Essential, New Delhi. Arabic gum was purchased from Jumbo Trading, Thailand. Low molecular weight chitosan (LWCS) is derived from shrimp shells [33]. The chemicals were CH_3_COOH (Merck, Germany), Polysorbate Tween 80 (Sigma-Aldrich, Singapore), Sodium Tripolyphosphate (STPP) (CV. Progo Mulyo, Yogyakarta), Ethanol (Merck, Germany), and Hexane (Merck, Germany).

## 3. Methods

### 3.1. Preparation of Saffron Essential Oil Nanoparticle

Saffron essential oil (SEO) nanoparticle was made according to previously described procedures by Ahmadi et al. [34], with some modification. In brief, several amounts of LWCS were dissolved in 25 mL of acetic acid solution (1% *v*/*v*) under magnetic stirring for 10 min at 1500 rpm at room temperature. Then, several amounts of Arabic gum were dissolved in 25 mL of distilled water, respectively, under magnetic stirring for 30 min at 1500 rpm at room temperature until homogenous. About 1 mL of polysorbate (Tween® 80) as an emulsifier for EO was added to the chitosan solution and allowed to stir for 10 min at room temperature to obtain a homogeneous solution. Then, several amounts of SEO were dropped into the chitosan/Tween 80 solution and stirred for 30 min at 700 rpm at room temperature. Subsequently, Arabic gum was incorporated into the solution dropwise. Agitation continued at the same rate for 30 min at room temperature. The sodium tripolyphosphate (STPP) solution was separately prepared by dissolving 0.04 g of TPP in 10 mL of distilled water. Finally, the TPP solution was slowly added to the previous solution by stirring at 700 rpm for 10 min at room temperature. After that, the nanoparticle suspension sample was analyzed for *z*-average, polydispersity index (PDI), and zeta potential using the Nano Zetasizer (Malvern Zetasizer Nano Series Ver 6.20, Malvern Instruments Ltd., UK).

### 3.2. Spray Drying

The SEO nanoparticle dispersion was spray-dried with spray drying (Lab-Plant SD-05, Keison Products, UK) at inlet temperature 140°C, outlet temperature 100°C, with flow rate 5.1 mL/min. The dried powder was collected and stored in dark bottles and air-tight containers at 4°C until further analysis.

## 4. Optimization of Saffron Essential Oil Nanoparticle Using Response Surface Methodology with Box-Behnken Design

Response surface methodology with Box-Behnken design was used to investigate the effects of three independent variables: LWCS concentration, Arabic gum concentration, and ratio (core: wall material) on the responses of *z*-average, polydispersity index (PDI), and zeta potential of the SEO nanoparticles. The three independent variables were investigated at three levels (-1, 0, 1) obtained from the preliminary assay. The coded and uncoded variables were used in Table 1.

The saffron essential oil nanoparticles were fitted using a second-order polynomial equation, and multiple regression of the data was carried out for obtaining an empirical model related to the most significant factors. The general form of the second-order polynomial equation is referred to Equation (1).

(1) $$\begin{array}{c} .Y=\beta_{0}+\sum \beta_{i}x_{i}+\sum \beta_{ii}{x_{i}}^{2}+\sum \beta_{ij}x_{i}x_{j}. \end{array}$$

*Y* is the predicted response, *x*_*i*_ and *x*_*j*_ are independent factors, *β*_0_ is the model intercept, *β*_*i*_ is the linear coefficient, *β*_*ii*_ is the quadratic coefficient, and the *b*_*ij*_ is the interaction coefficient. The response variable was then analyzed using ANOVA. The model that gives significance to ANOVA and nonsignificance to the lack of fit is chosen to analyze the variables.

### 4.1. Verification of RSM Model

Verification of the optimization result model is done by remaking the saffron essential oil nanoparticles according to the optimum conditions obtained from the program. Verification was carried out with two repetitions. The results obtained were compared with the value of the response variable predicted by RSM. The verification was then strengthened through an analyze comparison of mean one-sample *t*-test using SPSS with a confidence level of 95%. The prediction is declared successful if the verification results are close to or exactly the prediction value (*p* value > 0.05).

### 4.2. Encapsulation Efficiency

Crocin substances were used as the parameter of efficiency. There were two analyses to determine the encapsulation efficiency, namely, the total trapped crocins and the total crocins on the surface of the encapsulated product.

#### 4.2.1. Total Crocins

A modified method based on Bagheri et al. [35], with some modifications, was used to calculate total crocin SEO nanoparticles. A total of 0.5 g powder sample was dispersed with 40 mL distilled water in a flask, and this mixture was homogenized for 30 min: after which, 40 mL ethanol-hexane (4 : 3, *v*/*v*) was added, and the solution was homogenized for 10 min. Then, centrifuged for 10 min at 4000 rpm (25°C). The absorption of total crocin was determined at 440 nm with spectrophotometer UV-Vis (Shimadzu Uv 1601, Kyoto Jepang). Each measurement was carried out in triplicate. Quantification of total crocins was accomplished using crocin standard calibration curves (*y* = 0.52*x* − 0.031, with *R*^2^ = 0.99).

#### 4.2.2. Surface Crocins

A modified method based on Bagheri et al. [35], with some modification, was used to calculate surface crocin SEO nanoparticles. A total of 0.5 g powder sample was dispersed with 40 mL ethanol-hexane (4 : 3, *v*/*v*) in a flask, and this mixture was homogenized for 30 min. Then, the solution was centrifuged for 10 min at 4000 rpm (25°C). The absorption of surface crocin in the upper phase was determined at 440 nm with spectrophotometer UV-Vis (Shimadzu Uv 1601, Kyoto Jepang). Each measurement was carried out in triplicate. Quantification of surface crocins was accomplished using crocin standard calibration curves (*y* = 0.52*x* − 0.031, with *R*^2^ = 0.99).

#### 4.2.3. Encapsulation Efficiency

The encapsulation efficiency (%) of crocins was calculated using the following equation:

(2) $$\begin{array}{c} \text{EE}\%:\frac{\text{Total crocin}‐\text{Surface}\text{crocin}}{\text{Total crocin}}\times 100\%. \end{array}$$

#### 4.2.4. Scanning Electron Microscope (SEM)

The morphology of nanoparticles was assessed using a *Scanning Electron Microscope* (SEM) (JED 2300, Japan). For SEM, sample preparation was done by the nanoencapsulated powder sprinkled onto a two-sided adhesive tape and then coated with a thin layer of gold and observed with an accelerated voltage of 320 kV and photographed at 10000x.

### 4.3. FTIR

The structure analysis of the samples was examined by Fourier transform infrared (FTIR) (Thermo Nicolet Avatar 370 FT-IR). The samples were crushed and blended with potassium bromide (KBr) and pressed using a manual pellet press. FTIR spectra were scanned and recorded in the wavenumber range of 4000 to 400 cm^−1^.

## 5. Results and Discussion

### 5.1. Fitting the Models

Optimization is the process of finding the value of a considered optimal, effective, and efficient variable to achieve the desired results. The desired results were the saffron essential oil nanoparticles with the smallest *z*-average value (<100 nm), the polydispersity index (<1), and the maximum zeta potential value (<-30 mV; > +30 mV). The optimization of nanoparticles was carried out using the response surface methodology with the Box-Behnken design.

Statistical analysis (ANOVA) results in Table 2 revealed that the experimental data could be represented well with a quadratic polynomial model with a coefficient of determination *R*^2^ values for *z-average* and PDI dan zeta potential being 0.99, 0.9928, and 0.9686, respectively (Table 2), which means that the contribution of LWCS concentration, Arabic gum concentration, and ratio (oil: Arabic gum/LWCS) is 99% for the *z-average value*, 99.28% for the PDI value, and 96.86% for the zeta potential value. At the same time, the rest is influenced by other variables that are not included in the model as independent variables. If the value of *R*^2^ is closer to 1, it is an indication of a better model fitting to actual data [27]. High *R*^2^ values indicated that the quadratic model was highly efficient for fitting the data under the condition of the experiment [36].

The significant level for the quadratic polynomial model coefficient was determined through ANOVA (see Table 2). Quadratic model is significant with a *p* value < 0.05, namely, 0.002 for *z-average* response, <0.0001 for PDI, and<0.0030 for zeta potential. It can be said that the selected model can be used to describe well the relationship between the variables on the response observed. The lack of fit was nonsignificant (*p* value > 0.05) relative to pure error for all response variables, indicating that the model fitted well.

### 5.2. Effect of Independent Variables on Response

There were 15 runs with the predicted value of responses shown in Table 3 and ANOVA of all responses shown in Table 4.

### 5.3. *Z*-Average

The *z-average* measurement is aimed at determining the size distribution of saffron essential oil nanoparticles and at ensuring that the nanoparticles produced are nanometers (10^−9^ m) in size. Based on the data in Table 3, saffron essential oil nanoparticles have size ranges from 16.24 nm to 23.38 nm. This size is smaller than the result of Tan et al. [37] study that develops curcumin nanoparticles using chitosan and Arabic gum with an ionic gelation method. The developed nanoparticles showed the average diameter in the range of 250-290 nm. This is because of the difference between the molecular weight of saffron essential oil and curcumin.

Based on ANOVA data (Table 4), the concentration of LWCS and Arabic gum had a significant effect because they had *p* values of 0.01280 and 0.003 (<0.05). Meanwhile, the ratio (oil: Arabic gum/LWCS) did not significantly affect the *z*-average value *p* value of 0.4075 (>0.05). The interaction between the Arabic gum concentration and the LWCS concentration and the interaction between the Arabic gum and the ratio (oil: Arabic gum/LWCS) also had a significant effect with *p* values of 0.0426 and 0.0050 (<0.05), respectively. Meanwhile, the interaction between the LWCS concentration and the ratio (oil: Arabic gum/LWCS) did not significantly affect because the *p* value was >0.05. A larger *F* value and a smaller *p* value indicated a highly significant effect on response variables [38].

The equation obtained from the quadratic model for the *z*-average response is as follows:

(3) $$\begin{array}{c} Y=19.45+0.6322A+1.51B-0.1087C-0.4600AB-0.0706AC-0.2260BC-1.52A^{2}+1.91B^{2}-0.0815C^{2}, \end{array}$$

where *A* is the LWCS concentration, *B* is the Arabic gum concentration, and *C* is the ratio (oil: Arabic gum/LWCS).

Equation (3) shows that the increase in the *z-average* response value is due to the increasing LCWS and Arabic gum concentration indicated by a positive constant value. Therefore, more electrostatic interactions between the chitosan amino group (-NH3^+^) and the Arabic gum carboxyl group (-COO-) form a polyelectrolyte complex that produces larger nanometer-sized particles [39]. The response of the *z-average* value will also decrease as the ratio (oil: Arabic gum/LWCS) increases. According to Hadidi et al. [40], increasing chitosan/TPP content causes decreasing the sizes of nanoparticles.

### 5.4. Polydispersity Index (PDI)

Polydispersity index (PDI) is a value that shows the ratio between the average molecular mass weight and the average number of molecules where the value range is between 0 and 1. PDI demonstrates the homogeneity of particle size distribution and is one of the most critical formulation aspects in systems containing fine particles [41]. The values near zero indicate the higher homogeneity, and the values higher than 0.5 show the heterogeneity of particle size distribution [42].

Based on the data in Table 3, the average PDI value obtained is 0.495 to 0.607. This shows that the saffron essential oil nanoparticles produced have heterogeneity of particle size distribution so that they tend to aggregate one to each other [43].

The ANOVA data shows (Table 4) that LWCS concentration and ratio (oil: Arabic gum/LWCS) had a significant effect with *p* values of 0.0183 and 0.0185 (<0.05). Meanwhile, the Arabic gum concentration did not significantly affect the PDl value with a *p* value of 0.2204 (>0.05). The interactions occurring between the three independent variables (Arabic gum concentration, LWCS concentration, and ratio (oil: Arabic gum/LWCS)) also have a significant effect with *p* values of 0.0033, 0.0459, and 0.0264 (<0.05), respectively. A larger *F* value and a smaller *p* value indicated a highly significant effect on response variables [44].

The equation obtained from the model for the PDI response is presented as follows:

(4) $$\begin{array}{c} Y=+0.5515+0.0085A-0.0035B-0.0061C-0.0132AB-0.0019AC+0.0022BC+0.0369A^{2}-0.0268B^{2}-0.0007C^{2}, \end{array}$$

where *A* is the LWCS concentration, *B* is the Arabic gum concentration, and *C* is the ratio (oil: Arabic gum/LWCS).

Equation (4) shows that the PDl response value will increase directly proportional to the addition of the LWCS concentration and indicated by a positive constant value. The response of the PDl value will also decrease as the Arabic gum and the ratio (oil: Arabic gum/LWCS) increase. In contrast with Mohammadpour Dounighi et al. [45], PDI decreased as chitosan concentration was raised in reaction with a constant amount of TPP. As mentioned earlier, the reason behind this contradiction could be the difference between TPP and Arabic gum, the target material, and the ratio between chitosan and polyanions.

#### 5.4.1. Zeta Potential

Zeta potential is an important parameter to characterize the surface properties of nanoparticles and is related to electrostatic interactions between particles in colloid systems. The zeta potential value is also used to determine physical stability [46]. A good zeta potential value is within the range of <-30 mV or >30 mV. The higher the zeta potential value, the bigger the repulsive force between the particles due to charge. This then causes the particles to move randomly, and the dispersion system is stable.

Based on Table 3, the zeta potential value between -12 mV and -15.76 mV is still low. Thus, it has not reached the desired value (<-30 mV or >30 mV). The product is still less stable and tends to aggregate. It is presumably due to the weak repulsion between the particles [25]. Dispersion stability occurs when the electrostatic repelling forces between particles are more dominant than their attractive forces. Therefore, it can prevent agglomeration under certain conditions. According to Feyzioglu and Tornuk [47], chitosan nanoparticles loaded with different summer savory essential oil levels had negative zeta potential values varying from -7.54 to -21.12 mV. The sign (-) in the zeta potential value means that the saffron essential oil nanoparticle dispersion system negatively charges. This negative charge indicates that all (-NH3^+^) LWCS groups have been neutralized by the negative charge from Arabic gum and the STPP cross-linker used. In addition, a negative value on the zeta potential indicates that the nanoparticles are present in the matrix [48].

The Arabic gum concentration significantly affects changes in the zeta potential value with a *p* value of 0.0004 (<0.05) based on ANOVA data (Table 4). Meanwhile, the LWCS concentration and the ratio (oil: Arabic gum/LWCS) did not have a significant effect with a *p* value of >0.05. The interaction between the Arabic gum concentration and the LWCS concentration and the interaction between the Arabic gum concentration and the ratio (oil: Arabic gum/LWCS) also had a significant effect with *p* values of 0.0260 and 0.0036 (<0.05), respectively. Meanwhile, the interaction between the LWCS concentration and the ratio (oil: Arabic gum/LWCS) did not significantly affect with a *p* value of >0.05.

The equation obtained from the model for the zeta potential response is presented as follows:

(5) $$\begin{array}{c} Y=14.25+0.0995A-1.32B-0.0875C+0.5000AB-0.1022AC+0.2300BC+0.8625A^{2}-0.8625B^{2}+0.0368C^{2}, \end{array}$$

where *A* is the LWCS concentration, *B* is the Arabic gum concentration, and *C* is the ratio (oil: Arabic gum/LWCS).

Equation (4) shows that the zeta potential response value will increase directly proportional to the addition of the LWCS concentration and the Arabic gum concentration and indicated by a positive constant value. The zeta potential response value will also decrease as the Arabic gum concentration and the ratio (oil: Arabic gum/LWCS) increase.

**Figure 1:**
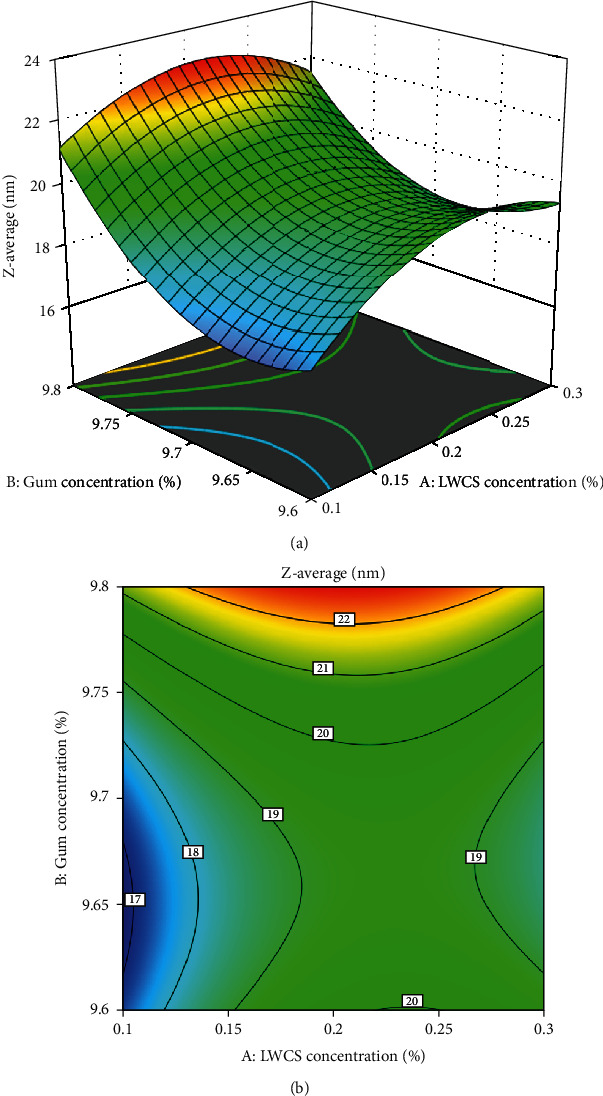
Surface plot (a) and contour plot (b) for *Z*-average response.

**Figure 2:**
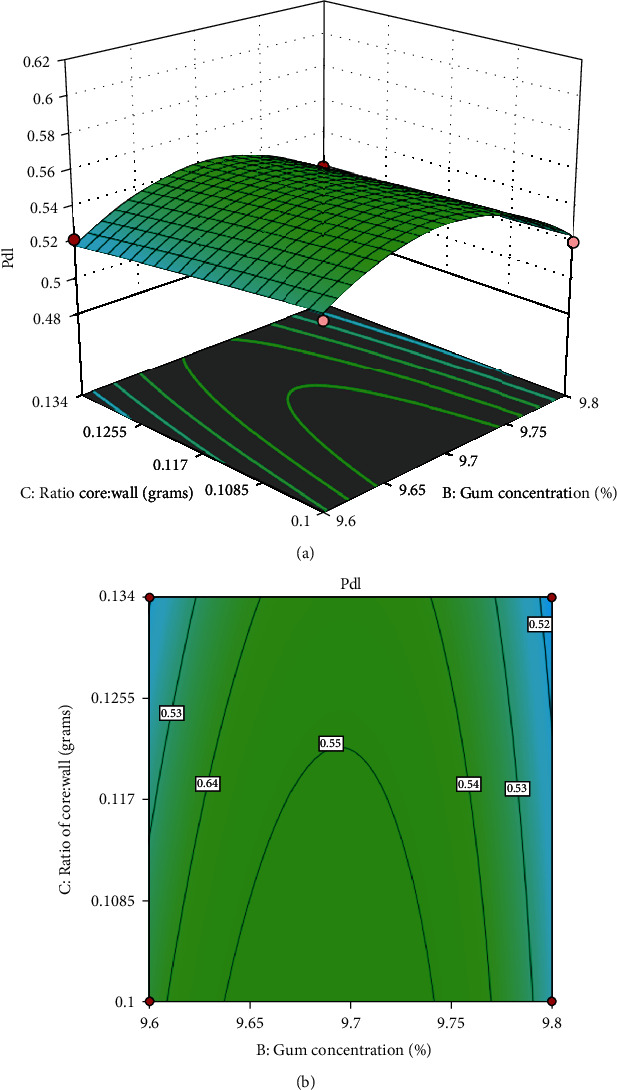
Surface plot (a) and contour plot (b) for PDI response.

**Figure 3:**
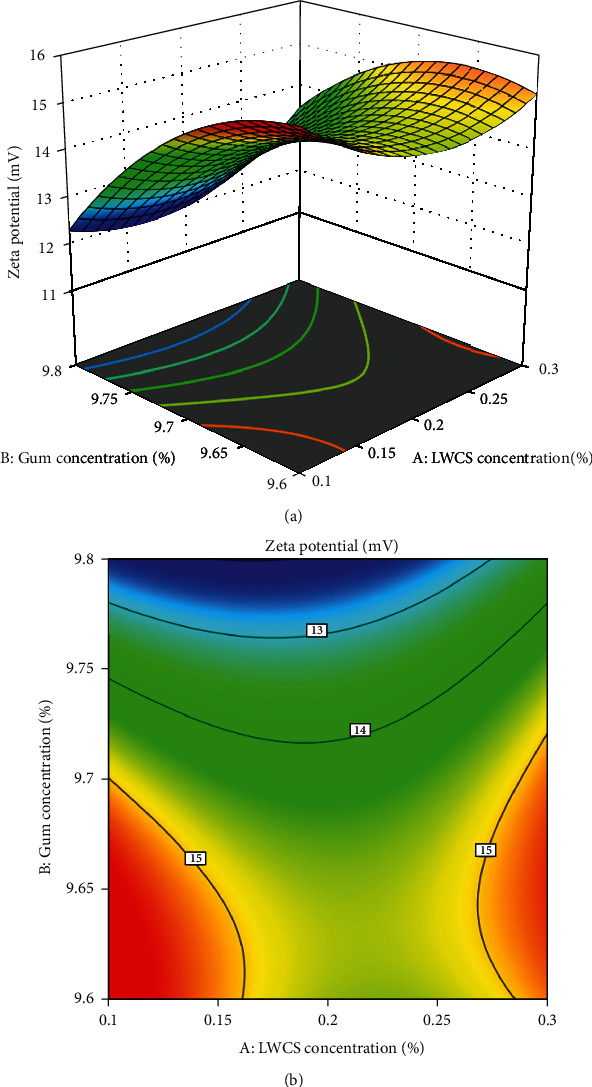
Surface plot (a) and contour plot (b) for zeta potential response.

The experimental data collected from the responses of the independent variables were analyzed. The results showed that the quadratic polynomial equation could illustrate the response surface plots and forecast the *z*-average, PDI, and zeta potential of the SEO nanoparticle. Figures 1–3 show the response surface and contour plots of the quadratic polynomial models that present the effect of independent variables on the *z*-average, PDI, and zeta potential of the SEO nanoparticles.

The concentration of LWCS and Arabic gum is a very important parameter that actively affects the *z*-average. The increase in the *z-average* response value is due to the increasing LCWS and Arabic gum concentration. Therefore, more electrostatic interactions between the chitosan amino group (-NH3^+^) and the Arabic gum carboxyl group (-COO-) form a polyelectrolyte complex that produces larger nanometer-sized particles.  Figure 1 shows that the minimum *z*-average value (16.24 nm) was achieved at LWCS concentration of 0.1% and Arabic gum concentration at 9.6%. The ratio (oil: Arabic gum/LWCS) had a significant effect on PDI response. The response of the PDl value will increase as the ratio (oil: Arabic gum/LWCS) increases.  Figure 2 shows that the minimum PDI value (0.495 nm) was achieved at the ratio (oil: Arabic gum/LWCS) of 1 : 5. The zeta potential response value will increase directly proportional to the addition of the Arabic gum concentration.  Figure 3 shows that the maximum zeta potential value (-15.8) was achieved at the Arabic gum concentration of 9.6%.

### 5.5. Optimization and Verification Formula of SEO Nanoparticles

**Figure 4:**
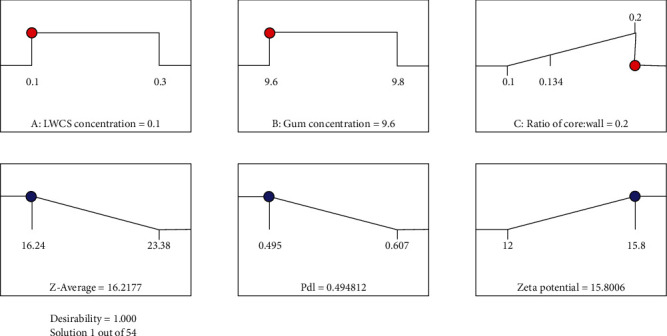
The optimum formulation from *Design Expert 12®* program.

The Design Expert 12® program provides 54 formula composition solutions based on determining the importance of each response. The chosen formula is the highest desirability value. The desirability value close to 1 indicates the closeness between the actual value and the predicted value. The optimum formula suggested by the program is the LWCS concentration of 0.1% and Arabic gum concentration of 9.6%, and the ratio (oil: Arabic gum/LWCS) was 1 : 5 with the desirability value of all factors was 1 (see Figure 4).

The optimum formula obtained from RSM was further confirmed by experimenting with the optimum condition. The optimum formula is predicted to produce saffron essential oil nanoparticles with a *z-average* value of 16.2177 mV, PDI of 0.4948, and a zeta potential value of -15.8 (see Figure 4).

The verified result of the optimum formula obtained *z*-average values of 16.24, PDI of 0.495, and zeta potential of 15.76 (Table 5). Compared with the predicted value, the verification result value is in the range of 95% PI low and 95% PI high (Table 5). This result means that the chosen formula recommended by the Design Expert program is adequately good. Verification was then strengthened by the one sample *t*-test using SPSS. The results showed that the values were not significantly different (*p* value > 0.05). The experimental data is closer to the predicted value. It can be concluded that the RSM models could be used to study the quadratic effects of LWCS concentration, Arabic gum concentration, and ratio (oil: Arabic gum/LWCS) on the *z-average*, PDI, and zeta potential. Therefore, the application of the RSM with Box-Behnken was suitable for optimizing the saffron oil nanoparticles with desirable responses.

### 5.6. Encapsulation Efficiency

The total crocins, surface crocins, and encapsulation efficiency for SEO nanoparticles are shown in Table 6. The total crocin value indicated the amount of all crocins in the inner and surface of SEO nanoparticles. The surface crocin value indicates the number of crocins on the surface of the nanoparticle wall. Surface crocins are undesirable because crocins are very easily damaged if exposed to certain oxidants such as air, light, and water [49, 50].

Based on the data in Table 6, the saffron essential oil nanoparticles have an encapsulation efficiency value of 86.4%. The value of the encapsulation efficiency in this study is considered high. Atefi et al. [51] showed similar results, where the encapsulation efficiency of saffron essential oil with Arabic gum and ß-cyclodextrin ranged 72.49-90.51%. Rajabi et al. [15] showed that a mixture of a saffron extract with maltodextrin, Arabic gum, and gelatin-coating had a slightly higher encapsulation efficiency (41.95-91.01%).

Combining two coatings with different charges causes the two polymers to interact ionically to form a polyelectrolyte complex bond [52]. The effect of the polyelectrolyte complex bond between chitosan and Arabic gum will further strengthen the nanoparticle walls to reduce the release of SEO to the surface. Another factor that strengthens the nanoparticle wall is STPP, which encourages the cross-link reaction between the LWCS from NH3^+^ and the P_3_O_5_^−10^ functional group from STPP [53]. According to Jafari et al. [54], the encapsulation efficiency is influenced by the drying conditions, emulsion, bioactive compound characteristics, and wall material properties. The drying temperature is directly proportional to the evaporation rate and inversely proportional to the final water content of the dried microparticles. At high drying temperatures, there is a higher evaporation rate of water on the droplet surface, which leads to the rapid formation of a semipermeable membrane, resulting in the protection of the release of the bioactive compounds during the drying process and, consequently, in higher bioactive retention [55]. Wall material concentration is also a factor that affects the retention of the bioactive compounds due to their viscosity properties in the feed solution. The increase of solid content in the feed solution can increase bioactive retention [56–58].

### 5.7. Scanning Electron Microscope (SEM)

**Figure 5:**
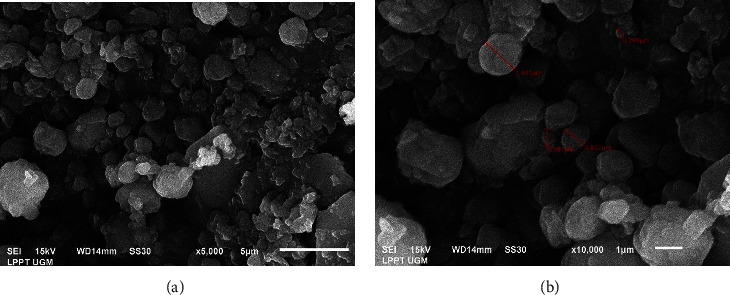
SEM image for saffron essential oil nanoparticles: (a) 5000x; (b) 10.000x.

Observation of the morphological profile of the nanoparticles was carried out using Scanning Electron Microscope (SEM). The observations in Figure 5 show that the nanoparticles are spherical, but some parts are shrinking/deflating. Most of the particles did not present a significant incidence of cracks or fissures in the outer surface, indicating a resistant external physical structure.

A similar morphology is also shown by Harris et al. [59], where the microcapsules produced are round and wavy due to the rapid evaporation of solvents during the spray drying process. According to Mohammed et al. [57], higher drying inlet (>140°C) temperatures tend to produce particles with a smoother surface. They found nanoparticles morphologically more defined and smoother, without evident cracks or particle agglomerations in the spray drying process both at 160 and 180°C. This fact may be attributed to rapid water evaporation and higher pressure inside the particles during microencapsulation at higher temperatures preventing shrinking. On the other hand, water diffusion is slower at lower temperatures, allowing more time for the particles to deform, wrinkle, and collapse.

Observation of the morphological profile using SEM is only able to see the shape of the surface of the nanocapsules, but the inside of the nanocapsules is not visible, so it is not possible to know the type of encapsulation, either the reservoir form (single-core) or matrix type (matrix core). To better know the type of encapsulation, it is necessary to observe the morphological profile using an infrared scanning electron.

### 5.8. FTIR

**Figure 6:**
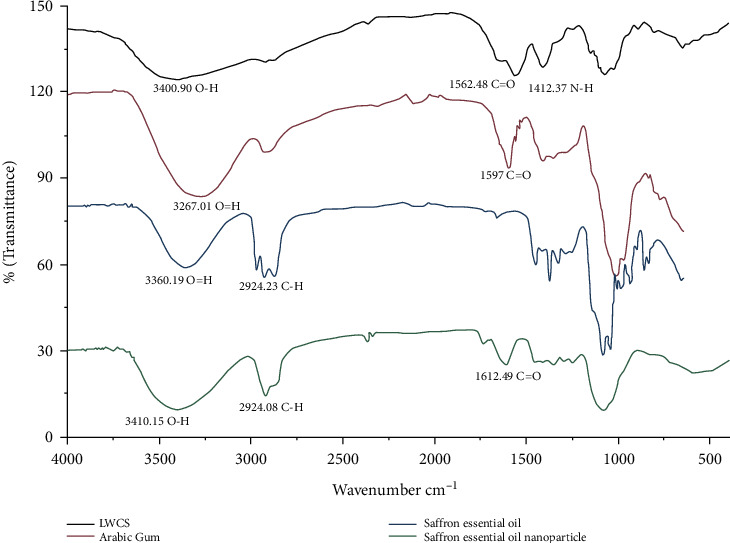
FTIR of saffron essential oil nanoparticles.

FTIR was used to confirm the linkage between (NH3^+^) and (COO-) of LWCS and Arabic gum, respectively, in the SEO nanoparticles (Figure 6). In the LWCS spectra, the broadband overlapping at 3400.90 cm^−1^ was concerned with the O-H groups. The strong band at 1562.48 was associated with the amide III groups (-NH3^+^). These peaks are typical peaks indicating the presence of an amine group on the LWCS. In addition, Arabic gum also shows a stretching vibration absorption C=O in wave number 1638.2 cm^−1^.

In the spectra of the SEO nanoparticles, the peaks for N-H bending vibration of amine 1412.37 cm^−1^ shifted to 1451.8 cm^−1^, and O-H stretching vibration peaks in wave number 3400.90 cm^−1^ shifted to 3410.15 cm^−1^ (Figure 6). These results indicate the interaction between amino groups of LWCS and (COO-) of Arabic gum. We can conclude that the appearance of these peaks is an indication of nanoparticle formation and that the inter- and intramolecular actions are enhanced in chitosan nanoparticles.

The formulation of saffron essential oil nanoparticles is produced through an ionic gelation reaction, which begins with the encapsulation interaction between LWCS and saffron essential oil. After encapsulation of saffron essential oil in the LWCS matrix, a nanoparticle will be formed. Stabilization occurs with the addition of Arabic gum. The (COO-) Arabic gum group will bind with the (NH3^+^) group of LWCS to form a polyelectrolyte complex bond [60]. As a result, there will be a change in the absorption peak in the IR spectra of the amino group, carboxyl group, and amide group. The strong electrostatic interaction between chitosan and a polyanion causes the shift or loss of distinct groups is caused by [61]. Mukhopadhyay et al. [62] reported a shift in the absorption peak of insulin nanoparticles encapsulated by the chitosan-alginate matrix as polyanions. A shift in the O-H and N-H chitosan stretching vibration peaks in wave numbers 3427 cm^−1^ to 3469 cm^−1^ in the insulin nanoparticle spectra.

## 6. Conclusions

Saffron essential oil nanoparticles were successfully prepared via the ionic gelation method through RSM with Box-Behnken design. A quadratic polynomial model was statistically significant (*p* value < 0.05). The quadratic model can be used to describe well the relationship between the variables on the response observed. The lack of fit was nonsignificant (*p* value > 0.05) relative to pure error for all response variables, indicating that the model fitted well. The model equation obtained for the process through RSM was adequate. The LWCS concentration and Arabic gum concentration had a significant effect on *z*-average and PDI. The ratio (oil: Arabic gum/LWCS) has a significant effect on zeta potential. The optimum formula was observed when LWCS concentration of 0.1%, Arabic gum concentration of 9.6%, and a ratio (oil: Arabic gum/LWCS) of 1: 5. The optimum formulas may produce saffron essential oil nanoparticles with a *z*-average value of 16.2177, a PDI value of 0.4948, and a zeta potential value of -15.8. The verification values were close to the predictive value given by the Design Expert® 12 program with (*p* value > 0.05) at the 95% confidence level. Therefore, the application of the RSM with BBD was suitable for optimizing the saffron oil nanoparticles with desirable responses.

## Figures and Tables

**Table 1:** Actual levels at coded factor levels of independent variables used in the RSM.

Independent variable	Symbol	Coded level		
		-1	0	+1
LWCS (%)	*X*_1_	0.1%	0.2%	0.3%
Arabic gum (%)	*X*_2_	9.6%	9.7%	9.8%
Ratio (oil: AG/LWCS)	*X*_3_	1 : 7.5	1 : 5	1 : 10

**Table 2:** Fitting the models.

Response	Models	*R* ^2^	Significant (*p* value < 0.05)	Lack of fit (*p* value > 0.05)
*Z*-average	Quadratic	0.9900	0.0002	0.1942
PDI	Quadratic	0.9928	<0.0001	0.1899
Zeta potential	Quadratic	0.9686	<0.0030	0.1707

**Table 3:** Experimental design with coded factor levels of independent variables and predicted values of responses.

Run	LWCS (%)	Arabic gum (%)	Ratio (oil: AG/LWCS)	*Z*-average (nm)	PDI	Zeta potential (mV)
1	(0)	(1)	(1)	22.2	0.52	-12
2	(-1)	(-1)	(0)	18.33	0.607	-15.2
3	(1)	(0)	(-1)	17.14	0.588	-14.8
4	(0)	(0)	(0)	18.7	0.586	-15.3
5	(0)	(0)	(0)	16.24	0.495	-15.8
6	(1)	(0)	(1)	17.04	0.508	-14.5
7	(-1)	(0)	(1)	17.25	0.57	-15.3
8	(-1)	(1)	(0)	17.14	0.503	-14.8
9	(0)	(1)	(-1)	19.86	0.532	-15.3
10	(1)	(-1)	(0)	23.38	0.521	-12.2
11	(0)	(-1)	(-1)	19.69	0.523	-14.2
12	(0)	(0)	(0)	16.76	0.503	-14.8
13	(0)	(-1)	(1)	17.68	0.52	-14
14	(-1)	(0)	(-1)	17.99	0.536	-14.4
15	(1)	(1)	(0)	17.59	0.508	-14.6

**Table 4:** ANOVA of *Z*-average, PDI, and zeta potential.

ANOVA	*Z*-average		PDI		Zeta potential	
Source	*F* value	*p* value	*F* value	*p* value	*F* value	*p* value
Model	55.21	0.0002	76.84	<0.0001	17.11	0.0030
*A*, LWCS %	14.37	0.0128	11.88	0.0183	0.4031	0.5534
*B*, gum %	82.26	0.0003	1.96	0.2204	71.32	0.0004
*C*, ratio oil: GA/LWCS	0.8171	0.4075	11.80	0.0185	0.5989	0.4740
*AB*	7.31	0.0426	27.60	0.0033	9.78	0.0260
*AC*	2.22	0.1963	6.98	0.0459	5.28	0.0700
*BC*	22.79	0.0050	9.71	0.0264	26.73	0.0036
*A* ^2^	73.31	0.0004	197.77	<0.0001	26.86	0.0035
*B* ^2^	116.49	0.0001	104.49	0.0002	26.86	0.0035
*C* ^2^	9.20	0.0290	3.20	0.1338	2.12	0.2051
Lack of fit	4.31	0.1942	4.42	0.1899	5.02	0.1707

**Table 5:** Predicted and verification value of *Z*-average, PDI, and zeta potential.

Response	Predicted	Verification	95% PI low	95% PI high	*p* value
*Z*-average	16.2177	16.24	15.215	17.2203	0.972
PDI	0.494812	0.495	0.479949	0.509676	0.987
Zeta potential	15.8006	-15.76	14.8582	16.7429	0.893

**Table 6:** Encapsulation efficiency of saffron essential oil nanoparticles.

Ulangan	Total crocin	Surface crocin	Encapsulation efficiency (%)
1	1.4076	0.1923	86.33%
2	1.4019	0.1884	86.56%
3	1.4057	0.1923	86.31%
$\bar{X}\pm \text{SD}$	1.4030	0.191	86.4%

## Data Availability

The data used to support the findings of this study are included in the article.
